# Automated Pharmacometric Model Development by Leveraging Low‐Dimensional Neural ODEs and LASSO Regression

**DOI:** 10.1002/psp4.70285

**Published:** 2026-06-24

**Authors:** Dominic, Stefan Bräm, Bernhard Steiert, Britta Steffens, Marc Pfister, Gilbert Koch

**Affiliations:** ^1^ Pediatric Pharmacology and Pharmacometrics University Children's Hospital Basel UKBB Basel Switzerland; ^2^ Roche Pharma Research and Early Development, Pharmaceutical Sciences, Roche Innovation Center Basel F. Hoffmann‐La Roche Ltd. Basel Switzerland

**Keywords:** automatic modeling, LASSO regression, machine learning, neural ordinary differential equations, pharmacometrics

## Abstract

Current pharmacometrics (PMX) model development is a manual process with iterative model building, fitting, and evaluation, which can be resource‐intensive and time‐consuming. Existing automated model development approaches utilize algorithms that still rely on iterative processes and perform model selection based on goodness‐of‐fit criteria. Recent advances in machine learning and artificial intelligence, particularly neural ordinary differential equations (NODEs), have demonstrated strong potential for characterizing complex pharmacokinetic (PK) and pharmacodynamic (PD) dynamics directly from data. However, NODEs are inherently black‐box models, which limits their interpretability and their ability to provide mechanistic insights, both of which are essential in PMX. We recently presented a promising concept that proposes interpretable ODE‐based structural models from NODEs but relied on manually identifying functional relationships, which can be challenging and prevents full automation. In this work, we present an automated model development approach that combines NODEs with least absolute shrinkage and selection operator (LASSO) regression to automatically propose structural models based on the dynamics learned by NODEs. The approach leverages LASSO's feature selection capability, thereby linking data‐driven modeling with interpretable mechanistic structures. We demonstrate the applicability of this automated NODE‐LASSO model development approach in three different scenarios: neonatal weight development, bi‐exponential PK data, and warfarin PK/PD data. The results indicate that our automated NODE‐LASSO model development approach can recover meaningful, mechanism‐based structures while reducing the need for extensive iterative and manual model development. This highlights its potential as a resource‐efficient and interpretable modeling strategy for PMX and its applications in model‐informed drug development and clinical research.

## Study Highlights

- What is the current knowledge on the topic?

The conventional pharmacometric model development approach is a stepwise process, where an initial structural model is iteratively adjusted based on goodness‐of‐fit metrics. This approach has also been adopted in current automation algorithms. Recent modeling approaches utilize machine learning, particularly neural ordinary differential equations (NODEs), to fit pharmacometric data without specifying a structural model with explicit, mechanism‐based equations.

- What question did this study address?

This work investigates whether NODEs can be utilized to propose a structural model with explicit, mechanism‐based equations in an automated way.

- What does this study add to our knowledge?

We present an approach based on NODEs and LASSO regression that can automatically identify explicit functions in an ODE that describes the dynamics in the data. Since this process is based on the dynamics learned by neural networks in a NODE and thus does not rely on solving any ODEs apart from the initial NODE fit, this approach is highly efficient.

- How might this change drug discovery, development, and/or therapeutics?

The presented approach helps to facilitate and accelerate pharmacometric model development.

## 1 Introduction

Pharmacometrics (PMX), as the field of quantitative analysis of pharmacokinetics (PK), pharmacodynamics (PD), and beyond, is an essential part of drug development and clinical research [1]. For pharmaceutical companies and medical agencies, model‐informed drug development (MIDD) has become an important pillar [2, 3, 4]. In MIDD and clinical research, PMX models are utilized, e.g., to inform clinical studies and to optimize treatment strategies.

PMX models typically utilize ordinary differential equations (ODEs) developed on observed PK and/or PD data. Currently, PMX model development is an iterative process where an initial structural model is fitted to the data [5], goodness‐of‐fit metrics are evaluated, and the model is adjusted accordingly. This iterative stepwise refinement approach can require substantial resources and time. One reason why identifying the structural model from PMX data is difficult is that while data is typically provided as a longitudinal state with observations at different time points, modeling is done in the derivative space, i.e., by characterizing the changes in the state per time, the so‐called right‐hand side of an ODE. For example, data from a one‐compartment PK model is seen as an exponential decay, but it is modeled with a linear function in the derivative space.

Several approaches have been introduced to automate the model development process [6, 7, 8, 9] by automating model selection and adjustment based on goodness‐of‐fit criteria. Thus, they are still based on a trial‐and‐error approach with iteratively fitting different standard PMX structural models to the data. Since solving ODEs numerically can be time‐consuming and finding a suitable structural model can require many iterations, especially if the dynamics in the data are complex, such automation approaches are often still inefficient.

Recent approaches for data fitting in PMX apply machine learning (ML) [10, 11], especially neural ODEs (NODEs) [12, 13, 14, 15, 16] which are ODEs with neural networks (NNs) on their right‐hand side. NNs are universal approximators, i.e., they can learn behaviors of data purely based on oberservations [17]. Several publications have shown the potential of NODEs to fit PMX data, demonstrating that NODEs can identify the underlying dynamics in the data [12, 13, 14, 15, 16] without the need of defining any explicit structural model.

While NNs and NODEs are excellent at fitting data without structural specifications, they are inherently black‐box models, meaning they provide limited insights into the functional relationships in the data. However, the understanding of the dynamics in a PMX model and the assessment of functional relationships is essential in PMX.

Recently, a concept to derive a conventional, ODE‐based structural model from a fitted NODE has been presented [18]. First, an NODE was fitted to PMX data, i.e., the NNs in the NODE learned the dynamics of the PMX data in the derivative space. Then, the output of the trained NNs, representing the derivative space, was visualized in so‐called derivative‐versus‐state‐plots allowing to manually identify explicit mechanism‐based functions that describe similar dynamics as the NNs have learned. The NNs are then substituted by these explicit functions. Finally, the proposed ODE is fitted to the PMX data. Thus, only an ODE is solved numerically in the first and the last step, whereas the model development, i.e., identifying explicit mechanism‐based functions that characterize the dynamics of the PMX data, does not include solving any ODE at all. Therefore, this approach circumvents the iterative model development process resulting in a substantial reduction in required resources and time. However, manual identification of a set of explicit functions can be challenging, especially when the dynamics are complex.

In this work, an entirely automated version of the model development approach just described is presented. To this end, the ML method least absolute shrinkage and selection operator (LASSO) regression [19] was applied. LASSO regression is commonly utilized for feature selection; hence it is well suited for automatically selecting a set of explicit functions that describe the dynamics in the derivative space learned by the NNs, which was performed manually in the previously mentioned work. This leads to a fully automated and numerically extremely efficient model development approach that does not require prior knowledge and is well suited to model PMX data with complex dynamics. The presented automated NODE‐LASSO model development approach was tested and evaluated for its applicability in different scenarios.

## 2 Methods

First, we present the general idea of the automated NODE‐LASSO model development approach and describe the required steps. Three example applications are presented to illustrate different scenarios, and applied software used in the implementation is summarized.

### 2.1 Automated NODE‐LASSO Model Development Approach

As mentioned previously, PMX data is typically provided as a longitudinal state, whereas modeling is done in the derivative space. The automated NODE‐LASSO model development approach builds on the idea that the derivative space, i.e., the right‐hand side of the differential equation, is represented by trained NNs in a fitted NODE, hence data describing the derivative space can be generated. This allows to perform LASSO regression in form of a highly efficient regularized generalized linear model [20] to automatically identify a set of explicit, mechanism‐based functions that can replace the NNs. As a result, we derive a structural model that characterizes the PMX data without any NNs.

Our automated NODE‐LASSO model development approach consists of four steps:

1. Fitting a low‐dimensional NODE to the PMX data to learn the dynamics in the derivative space.
2. Accessing the dynamics of the NNs in the NODE by generating derivative data
3. Leveraging LASSO regression to identify a set of functions that describe the dynamics of the derivative data.
4. Implementing identified functions to derive the structural model and fitting it to the PMX data.

We emphasize that only in step (1) and (4) a PMX data fit is performed. The model development itself, i.e., identifying mechanism‐based functions, is done in step (2) and (3) without solving ODEs.

**FIGURE 1:**
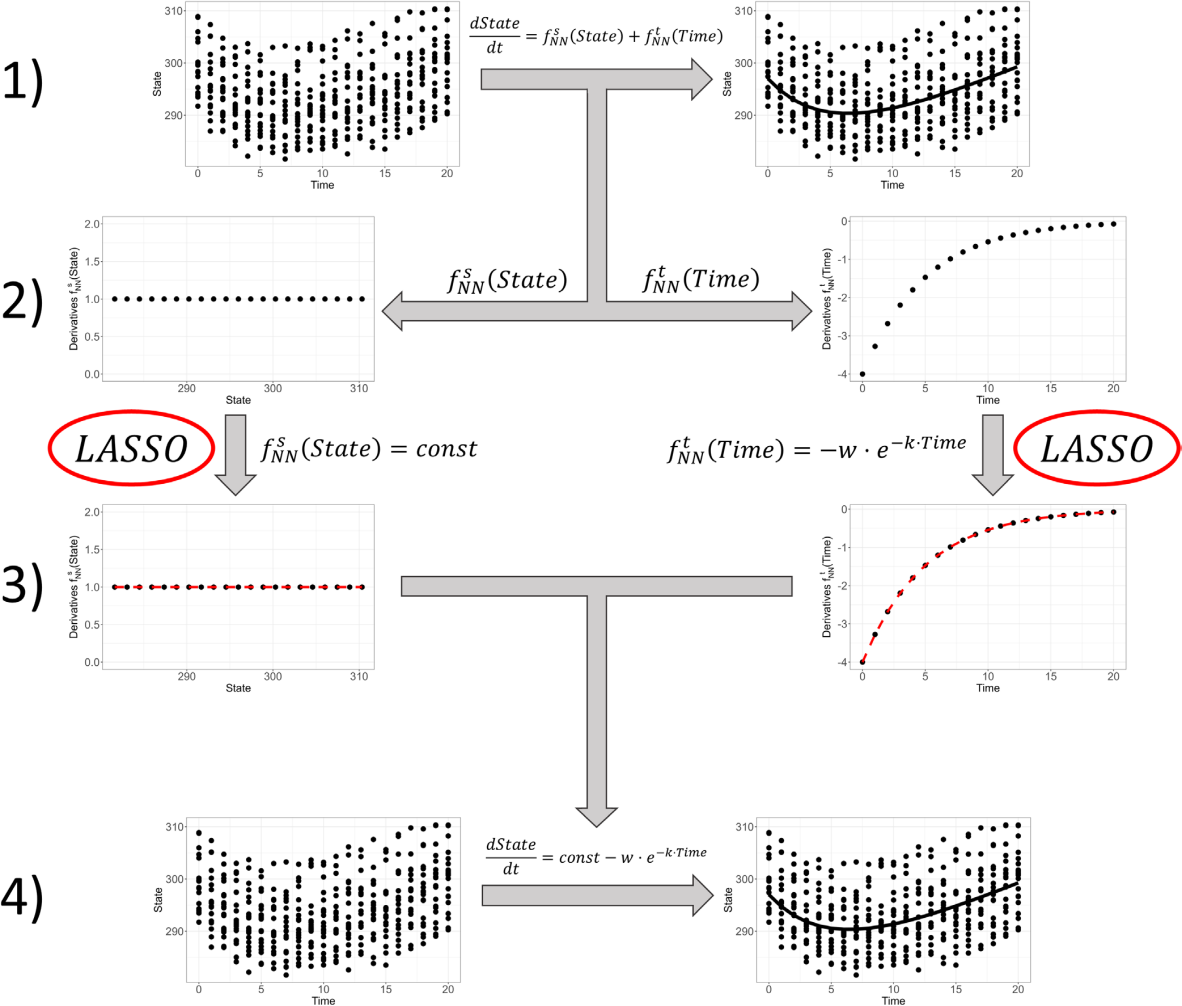
Overview of the general NODE‐LASSO approach with (1) fitting a low‐dimensional NODE to the data, (2) generating derivative data from the NNs, (3) utilizing LASSO regression to identify functions that describe derivative data, and (4) re‐implementing the identified functions in an ODE to fit the data.

A schematic of this general approach is shown in Figure 1. In the following, all four steps are explained in detail.

### 2.2 Detailed Explanation of the Four Steps

#### 2.2.1 Step 1: Fitting a Low‐Dimensional NODE to the PMX Data to Learn the Dynamics

Low‐dimensional NODEs are ODEs with one or multiple NNs on the right‐hand side of the differential equation ([14, 15]) and typically do not have unobserved/latent states such as peripheral compartments. The applied NNs in the NODEs have a one‐dimensional input and output, and one hidden layer with a small number of neurons. The standard form of low‐dimensional NODEs [14] reads as follows:

(1) $$\frac{\mathrm{dX}}{\mathrm{dt}}=f_{\mathrm{NN}}^{X}\left(X\right)+X_{0}\cdot f_{\mathrm{NN}}^{t}\left(t\right),\;X\left(0\right)=X_{0}$$

where X is the state to be modeled, $f_{\mathrm{NN}}^{X}$ a state‐dependent NN, $f_{\mathrm{NN}}^{t}$ a time‐dependent NN, and $X_{0}$ the initial value of the state. The multiplication of $f_{\mathrm{NN}}^{t}$ with $X_{0}$ is derived from the dimension reduction in low‐dimensional NODEs, explained in more detail in the original article [14]. However, we would like to highlight that other low‐dimensional NODE structures can be applied, depending on the type of data, modeling questions, and prior knowledge or assumptions about the underlying model.

Low‐dimensional NODEs were implemented in Monolix utilizing the *pmxNODE* package [14, 15] in R that was specifically developed to facilitate the application in pharmacometric software, such as Monolix, NONMEM, and the *nlmixr2* package [21, 22]. Architecture of NNs is described in the Supporting Information.

#### 2.2.2 Step 2: Accessing the Dynamics of the NNs in the NODE by Generating Derivative Data

To access the derivative space of a differential equation, so‐called derivative data is produced. More precisely, data that consists of the inputs to a function used on the right‐hand side of a differential equation and its corresponding outputs, i.e., the derivatives in the differential equation contributed by this function.

For illustration purposes, a simple one‐compartment PK model is considered

(2) $$\frac{dC_{C}}{\mathrm{dt}}=f\left(C_{C}\right),\;C_{C}\left(0\right)=\frac{D}{V},$$

where $C_{C}$ is the concentration in the central compartment, $f\left(C_{C}\right)$ the function on the right‐hand side, D the dose, and V the volume of distribution. In this example, the function on the right‐hand side is

(3) $$f\left(C_{C}\right)=-k_{\mathrm{el}}\cdot C_{C}.$$

**FIGURE 2:**
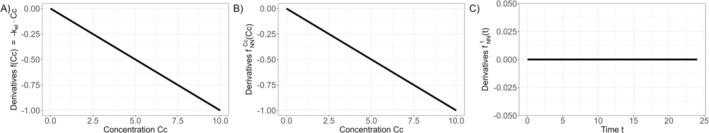
Visualization of derivative data for a one‐compartment model with (A) the mechanistic function $f\left(C_{C}\right)=-k_{\mathrm{el}}\cdot C_{C}$ plotted against concentration $C_{C}$, (B) the NN $f_{\mathrm{NN}}^{\mathrm{Cc}}\left(C_{C}\right)$ against $C_{C}$, and (C) the NN $f_{\mathrm{NN}}^{t}\left(t\right)$ against time t.

Thus, derivative data consists of a range of concentration as input and the predictions for $f\left(C_{C}\right)$ as output, as visualized in Figure 2A.

Due to the one‐dimensional character of NNs in the applied NODEs, the approach of derivative data can easily be translated to NNs in a low‐dimensional NODE Equation (1). To this end, the output of $f_{\mathrm{NN}}^{X}$ can be predicted for a range of state values and the output of $f_{\mathrm{NN}}^{t}$ for a range of time values. Such derivative data for NNs represent the dynamics that the NNs have learned from the data during fitting of the PMX data. If a low‐dimensional NODE Equation (1) was applied to fit data from a one‐compartment model, the state‐dependent NN $f_{\mathrm{NN}}^{X}$ would learn the linear function in the one‐compartment model and there would be no need that the time‐dependent NN $f_{\mathrm{NN}}^{t}$ contributes to the derivatives, thus being inactive, as illustrated in Figure 2B,C, respectively.

#### 2.2.3 Step 3: Leveraging LASSO Regression to Identify a Set of Explicit Functions

LASSO regression is a regression model with L1 penalization on coefficients for input variables. This means, coefficients for input variables not required to describe the response variable are estimated to be zero, i.e., only relevant input variables are selected. The L1 penalization and the consequential removal of input variables leads to a parsimonious proposed model, which is commonly the goal in PMX modeling.

The LASSO regression was performed on the derivative data generated from the fitted NNs in the NODE. The response variable was the derivatives. As input variables, the output of different candidate functions with different parameter sets were calculated for the state or time in the derivative data, later denoted as I. In this work, candidate functions correspond to typical functions commonly applied in PMX, i.e., constant, linear, exponential, and Emax functions with Hill coefficient, according to

(4) $$f_{\mathrm{Con}}\left(I\right)=v_{C}$$

(5) $$f_{\mathrm{Lin}}\left(I\right)=v_{L}\cdot I$$

(6) $$f_{\mathrm{Exp}}\left(I\right)=v_{E}\cdot e^{u_{E}\cdot I}$$

(7) $$f_{\text{Emax}}\left(I\right)=v_{\text{Emax}}\cdot \frac{I^{u_{h}}}{u_{E50}^{u_{h}}+I^{u_{h}}}.$$

Function parameters $u_{E}$, $u_{h}$ and $u_{E50}$ were equally spaced within a predefined range. More precisely, for $u_{E}$ and $u_{h}$, ranges were empirically set from −2 to 2, and 0 to 4, respectively, and the range for $u_{E50}$ depended on the range of inputs to the NN, see Table S1. In the LASSO regression, penalization was applied to account for numbers of parameters in the candidate functions, i.e., 1 for constant and linear, 2 for exponential, and 3 for Emax function. The function coefficients $v_{C},$ $v_{L}$, $v_{E}$ and $v_{\text{Emax}}$ were determined by the LASSO regression by optimizing the standard objective function value (OFV) from the *glmnet* [20] function in R according to

(8) $$\mathrm{OFV}=\frac{1}{2n}\mathrm{RSS}+\lambda \cdot {\left\|\beta \right\|}_{1}\;\;,$$

where *n* is the number of observations, *RSS* the residual sum of squares, λ the regularization parameter, and ${\left\|\beta \right\|}_{1}$ the L1 norm of the coefficient vector, i.e., $v_{C}$, $v_{L}$, $v_{E}$, and $v_{\text{Emax}}$.

If same functions with similar parameters were identified by the LASSO regression, these functions were summarized to one single function in order to find a parsimonious model without excessive flexibility and with well‐identifiable parameters when fitted to the actual data in step 4. For example, if two exponential functions with $u_{E1}=-0.1$ and $u_{E2}=-0.11$ were identified, one exponential function with initial estimate $u_{E}=-0.105$ was included in the identified set of functions. To account for the grouping, an adjusted equation for BIC was calculated according to

(9) $$\mathrm{BIC}=n\cdot \log\left(\frac{\mathrm{RSS}}{n}\right)+m\cdot \log\left(n\right)\;\;,$$

where m is the number of parameters in the grouped set of functions. A 10‐fold cross‐validation was performed from which $\lambda_{\mathrm{BIC},1\mathrm{se}}$, the largest λ where the cross‐validated BIC is within one standard error of the minimum BIC, was determined to identify a parsimonious set of functions.

#### 2.2.4 Step 4: Implementing Identified Functions to Derive the Structural Model

The identified set of functions from the LASSO regression were implemented in a structural model by replacing the NNs in the NODE. This means, if a LASSO regression was performed for the NODE in Equation (1) and the following sets of functions were identified:

$$f_{\mathrm{NN}}^{X}\left(X\right)\sim f^{1}\left(X\right)+f^{2}\left(X\right),$$

(10) $$f_{\mathrm{NN}}^{t}\left(t\right)\sim f^{3}\left(t\right)\;\;,$$

the proposed structural model was

(11) $$\frac{\mathrm{dX}}{\mathrm{dt}}=f^{1}\left(X\right)+f^{2}\left(X\right)+X_{0}\cdot f^{3}\left(t\right),\;X\left(0\right)=X_{0}.$$

When implementing the proposed structural model in Monolix, model parameters were assumed to be log‐normally distributed, as commonly done in PMX. Because estimated coefficients and parameters from the LASSO regression are not strictly positive, they were transformed using absolute values by $k=\left|v_{C}\right|$, $r=\left|v_{L}\right|$, $q=\left|v_{E}\right|$, $E_{\max}=\left|v_{\text{Emax}}\right|$, and $p=\left|u_{E}\right|$. The original sign of each coefficient or parameter was incorporated directly into the proposed model. For better readability, $u_{E50}$ and $u_{h}$ were renamed to E50 and h, respectively.

Performance of the proposed structural model was compared to the NODE in terms of mean absolute relative error (MARE) for predictions with and without random effects according to

(12) $$\text{MARE}=\frac{1}{n}\sum_{i=1}^{n}\frac{\left|\mathrm{Obs}-\text{Pred}\right|}{\mathrm{Obs}}\;\;,$$

where n is the number of observations, Obs are the observations, and Pred are either population or individual predictions. In addition, observation versus prediction plots are presented in Figure S3.

### 2.3 Example Applications

The presented automated NODE‐LASSO model development approach was tested in three applications for different datasets and observation types. In the first application, the approach was applied to characterize maturation‐related weight changes in newborns, to evaluate the general capability of LASSO regression to derive a structural model based on a low‐dimensional NODE. The second application represents a sensitivity analysis for characterizing bi‐exponential PK data originating from a two‐compartment model to assess whether the method can derive a reasonable model for data that originates from a model with a latent state, i.e., a peripheral compartment. The third application focuses on PD of warfarin to examine the potential of the method when applied selectively to a specific, unknown part of the initial model.

#### 2.3.1 Characterizing Maturation‐Related Weight Changes in Newborns

The weight dataset included 2425 neonates [23] each with a median of 5 [IQR: 4–6] observations in a period of 7 days. A one‐dimensional NODE to characterize weight progression was applied according to

(13) $$\begin{array}{c} \frac{\mathrm{dW}}{\mathrm{dt}}=f_{\mathrm{NN}}^{W}\left(W\right)+W_{0}\cdot f_{\mathrm{NN}}^{t}\left(t\right),\;W\left(0\right)=W_{0}\;\;, \end{array}$$

where W denotes the weight, t the time after birth, $W_{0}$ the birth weight, $f_{\mathrm{NN}}^{W}$ the weight‐dependent NN, and $f_{\mathrm{NN}}^{t}$ the time‐dependent NN.

#### 2.3.2 Characterizing Bi‐Exponential PK Data

PK data was simulated for 50 subjects with an intravenous two‐compartment PK model as

$$\frac{dA_{C}}{\mathrm{dt}}=-k_{\mathrm{el}}\cdot A_{C}-k_{12}\cdot A_{C}+k_{21}\cdot A_{P},\;A_{C}\left(0\right)=D,$$

$$\frac{dA_{P}}{\mathrm{dt}}=k_{12}\cdot A_{C}-k_{21}\cdot A_{P},\;A_{P}\left(0\right)=0,$$

(14) $$C_{C}=\frac{A_{C}}{V}.$$

For each subject, log‐normally distributed model parameters were sampled, and 7 concentration measurements were simulated over 24 h. Again, a one‐dimensional NODE according to

$$\frac{dA_{C}}{\mathrm{dt}}=f_{\mathrm{NN}}^{\mathrm{Ac}}\left(A_{C}\right)+D\cdot f_{\mathrm{NN}}^{t}\left(t\right),\;A_{C}\left(0\right)=D,$$

(15) $$C_{C}=\frac{A_{C}}{V},$$

was applied to fit this PK data where $A_{C}$ is the amount of the drug in the central compartment, $f_{\mathrm{NN}}^{A}$ and $f_{\mathrm{NN}}^{t}$ the amount‐dependent and time‐dependent NNs, respectively, D the dose, V the volume of distribution, and $C_{C}$ the concentration in the central compartment.

#### 2.3.3 Characterizing PD of Warfarin

The warfarin data (an example dataset in Monolix) included 32 patients receiving an oral single‐dose of warfarin, each with a median of 6 [IQR: 6‐10] Warfarin concentrations and 7 [IQR: 7‐8] prothrombin complex activity (PCA) observations. The NODE consisted of a mechanism‐based part for drug concentration modeling, i.e., a one‐compartment model with lag‐time in the absorption compartment, and an indirect response model for PCA. The production was described with a NN according to

$$\frac{dA_{A}}{\mathrm{dt}}=\text{In}\left(D,T_{\mathrm{lag}}\right)-k_{a}\cdot A_{A},\;A_{A}\left(0\right)=0,$$

$$\frac{dA_{C}}{\mathrm{dt}}=k_{a}\cdot A_{A}-k_{\mathrm{el}}\cdot A_{C},\;A_{C}\left(0\right)=0,$$

$$C_{C}=\frac{A_{C}}{V},$$

(16) $$\begin{array}{c} \;\frac{\text{dPCA}}{\mathrm{dt}}=f_{\mathrm{NN}}^{\mathrm{Cc}}\left(C_{C}\right)-k_{\text{out}}\cdot \mathrm{PCA},\;\mathrm{PCA}\left(0\right)=\mathrm{PC}A_{0}\;\;, \end{array}$$

where $f_{\mathrm{NN}}^{C}$ is the concentration‐dependent NN for the production.

### 2.4 Software

Data fitting with low‐dimensional NODEs and conventional models was done in Monolix 2021R2 [24]. The implementation of low‐dimensional NODEs in Monolix was done with the R package *pmxNODE* [14, 15]. Remaining analyses and visualizations were done in R [25] with *dplyr* [26] for data handling, *ggplot2* [27] for visualizations, and *glmnet* [19] for the LASSO regression.

## 3 Results

First, we present the fits with low‐dimensional NODEs for the three example applications. Then the three proposed structural models are presented, remarks of the findings are provided, and the fits are compared to the low‐dimensional NODE and, if applicable, to a previously developed reference PMX model in terms of MAREs.

### 3.1 Fitting a Low‐Dimensional NODE to PMX Data to Learn the Underlying Dynamics

**FIGURE 3:**
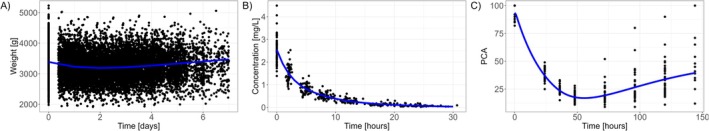
Observed data with NODE population fit for (A) the weight model, (B) the two‐compartment model, and (C) the warfarin model.

The low‐dimensional NODEs Equations (13), (15) and (16) fit the three example data, i.e., weight change data, two‐compartment data, and PD data of warfarin, well as shown for the population fits in Figure 3. No systematic errors can be observed in the goodness‐of‐fit plots, Figure S3. Since subsequent steps in the NODE‐LASSO modeling approach are depending on the NODE describing the dynamics in the data properly, it is essential to verify an adequate fit with the NODEs before continuing.

The MAREs for the population fit and the individual fits serve as benchmarks for the fits with the proposed structural model, i.e., to assess if the fitting capability of the proposed model is substantially lower than that of the NODE, see Table 1.

**TABLE 1:** Mean absolute relative errors (MARE) calculated from population (Pop) and individual (Ind) predictions with the NODE model, the proposed structural model, and if applicable a reference pharmacometrics model for the three examples.

Data	Prediction	MARE (NODE)	MARE (Proposed)	MARE (PMX)
Weight	Pop	0.092	0.092	—
	Ind	0.006	0.007	—
Two‐compartment	Pop	0.265	0.269	0.268
	Ind	0.089	0.097	0.088
Warfarin	Pop	0.238	0.217	0.221
	Ind	0.071	0.066	0.085

### 3.2 Proposed Structural Models

#### 3.2.1 Characterizing Maturation‐Related Weight Changes in Newborns

**FIGURE 4:**
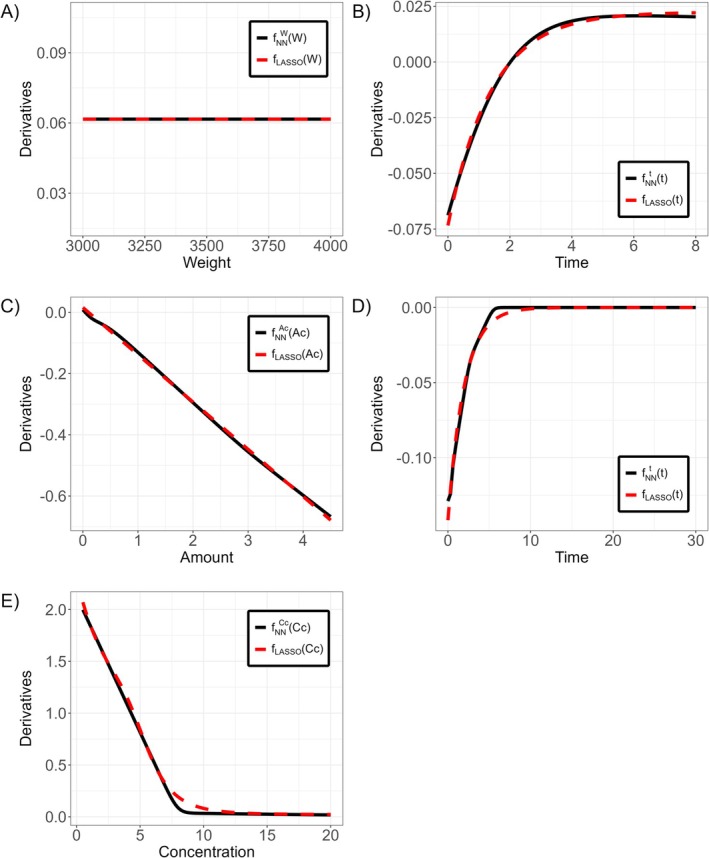
Derivative versus state/time plots for (A) $f_{\mathrm{NN}}^{W}\left(W\right)$ and (B) $f_{\mathrm{NN}}^{t}\left(t\right)$ in the weight NODE model, (C) $f_{\mathrm{NN}}^{\mathrm{Ac}}\left(A_{C}\right)$ and (D) $f_{\mathrm{NN}}^{t}\left(t\right)$ in the two‐compartment NODE model, and (E) $f_{\mathrm{NN}}^{\mathrm{Cc}}\left(C_{C}\right)$ in the warfarin NODE model with the derivative data from the NN as black solid line and the through LASSO identified function as red dashed line.

The identified sets of functions from the LASSO regression were

$$f_{\mathrm{NN}}^{W}\left(W\right)\sim f_{\mathrm{Con}}\left(W\right)=k_{W},$$

(17) $$f_{\mathrm{NN}}^{t}\left(t\right)\sim f_{\mathrm{Con}}\left(t\right)+f_{\mathrm{Exp}}\left(t\right)=k_{t}-q\cdot e^{-p\cdot t},$$

as visualized in Figure 4. This indicates a weight reduction that decreases over time, followed by a linear weight increase. Thus, the proposed structural model reads:

(18) $$\frac{\mathrm{dW}}{\mathrm{dt}}=k_{W}+W_{0}\cdot \left(k_{t}-q\cdot e^{-p\cdot t}\right),\;W\left(0\right)=W_{0}.$$

This model was fitted to the data and parameters $k_{w}$, $k_{t}$, q, p, and $W_{0}$ were estimated. Similar MAREs of the proposed structural model Equation (18) compared to the NODE Equation (13) indicate the successful translation of the NODE containing NNs to a conventional, interpretable ODE model, compare Table 1.

#### 3.2.2 Characterizing Bi‐Exponential PK Data

The identified sets of functions from the LASSO regression were

$$f_{\mathrm{NN}}^{\mathrm{Ac}}\left(A_{C}\right)\sim f_{\mathrm{Lin}}\left(A_{C}\right)=-r\cdot A_{C},$$

(19) $$f_{\mathrm{NN}}^{t}\left(t\right)\sim f_{\mathrm{Exp}}\left(t\right)=-q\cdot e^{-p\cdot t},$$

as visualized in Figure 4. This represents a biphasic drug elimination from the central compartment, one where elimination rate scales linearly with drug amount and one which is active after dosing but decreases with time. Thus, the proposed structural model is

$$\frac{dA_{C}}{\mathrm{dt}}=-r\cdot A_{C}-D\cdot q\cdot e^{-p\cdot t},\;A_{C}\left(0\right)=D,$$

(20) $$C_{C}=\frac{A_{C}}{V}.$$

The model was fitted to the data and parameters r, q, p, and V were estimated. Similar MAREs of the proposed structural model Equation (20) compared to the NODE Equation (15) were observed.

Additionally, the original two‐compartment PK model Equation (14) was fitted to the PMX data as reference, and MAREs were calculated. This demonstrates that the NODE Equation (15) and the proposed structural model Equation (20) fitted the data similarly well as the model utilized for data generation.

In the following, some remarks regarding the proposed structural model Equation (20) are provided. The explicit solution of Equation (20) reads:

(21) $$C_{C}\left(t\right)=A^{\prime }\cdot e^{-p\cdot t}+B^{\prime }\cdot e^{-r\cdot t},$$

with $A^{\prime }=-\frac{D}{V}\cdot \frac{w}{k-p}$ and $B^{\prime }=\frac{D}{V}\cdot \frac{k-p+w}{k-p}$, compare Supporting Information for derivation. Hence. Equation (21) is equal to the commonly known explicit solution of the central compartment in the standard two‐compartment PK model in Equation (14)

(22) $$C_{C}\left(t\right)=A\cdot e^{-\alpha \cdot t}+B\cdot e^{-\beta \cdot t},$$

with the macro parameterization A, B, α, and β. Also, macro parameter values in Equation (21) and (22) are similar when deriving from estimated parameters in Equations (20) and (14), respectively, compare Table S2. The similar explicit solution and proposed parameters additionally demonstrate the plausibility of the proposed structural model from the LASSO regression Equation (20) and its capability to describe dynamics in the central compartment similar to a two‐compartment PK model. Further analyses, including multiple dosing scenarios, are presented in the Supporting Information.

#### 3.2.3 Characterizing PD of Warfarin

The identified set of functions for $f_{\mathrm{NN}}^{\mathrm{Cc}}$ from the LASSO regression was

$$f_{\mathrm{NN}}^{\mathrm{Cc}}\left(C_{C}\right)\;\sim f_{\mathrm{Con}}\left(C_{C}\right)+f_{\text{Emax}}\left(C_{C}\right)+f_{\mathrm{Exp}}\left(C_{C}\right)$$

(23) $$\;=k-E_{\max}\cdot \frac{C_{C}^{h}}{E_{50}^{h}+C_{C}^{h}}+q\cdot e^{-p\cdot C_{c}},$$

as visualized in Figure 4. To ensure steady‐state behavior when no drug is given, Equation (23) was rearranged by introducing $I_{\max}=\frac{E_{\max}}{k}$ as well as $k_{\text{in}}=k+q$ and $w=\frac{q}{k_{\text{in}}}$. Hence, the proposed structural model reads:

$$\frac{dA_{A}}{\mathrm{dt}}=\text{In}\left(D,T_{\mathrm{lag}}\right)-k_{a}\cdot A_{A},\;A_{A}\left(0\right)=0,$$

$$\frac{dA_{C}}{\mathrm{dt}}=k_{a}\cdot A_{A}-k_{\mathrm{el}}\cdot A_{C},\;A_{C}\left(0\right)=0,$$

$$C_{C}=\frac{A_{C}}{V},$$

$$\begin{array}{c} \frac{\text{dPCA}}{\mathrm{dt}}=k_{\text{in}}\cdot \left(1-w\right)\cdot \left(1-I_{\max}\cdot \frac{C_{C}^{h}}{IC_{50}^{h}+C_{C}^{h}}\right)\\ \;+k_{\text{in}}\cdot w\cdot e^{-p\cdot C_{C}}-k_{\text{out}}\cdot \mathrm{PCA}, \end{array}$$

(24) $$\;\mathrm{PCA}\left(0\right)=\mathrm{PC}A_{0}\;\;,$$

where $k_{\text{in}}$ is the production rate calculated as $k_{\text{in}}=\mathrm{PC}A_{0}\cdot k_{\text{out}}$. Interestingly, the production rate is split by $\left(1-w\right)$ representing the typical $I_{\max}$ inhibition dependency on $C_{C}$, and w highlighting an additional $C_{C}$ related exponential part.

The proposed ODE in Equation (24) was fitted to the PD data and parameters were estimated. Due to high shrinkage observed in the initial data fit, random effects were removed from $I_{\max}$, h, and $IC_{50}$. Similar MAREs were observed, comparing the NODE model and the proposed ODE model, compare Table 1.

Additionally, the commonly applied Warfarin PKPD model [24] with only an inhibitory $I_{\max}$ function in the indirect response model, i.e, with *w* = 0 in Equation (24), according to

(25) $$\begin{array}{c} \begin{array}{c} \frac{\text{dPCA}}{\mathrm{dt}}=k_{\text{in}}\cdot \left(1-I_{\max}\cdot \frac{C_{C}}{IC_{50}+C_{C}}\right)-k_{\text{out}}\cdot \mathrm{PCA},\;\mathrm{PCA}\left(0\right)=\mathrm{PC}A_{0} \end{array}\;\;, \end{array}$$

was fitted to the data and higher MAREs were observed compared to the proposed model in Equation (24), see Table 1. Further, Equation (24) also resulted in a lower BIC (BIC = 2134) compared to the commonly applied Warfarin PKPD model (BIC = 2166).

## 4 Discussion

The automated NODE‐LASSO model development approach successfully proposed structural models capable of characterizing PMX data. Thus, our approach provides an alternative to the iterative modeling approach conventionally applied in PMX.

The initial fitting of the data with NODEs allows the unbiased identification of the dynamics in the data without the modeler having to propose an initial structural model. The application of NNs in the NODE further enables generating derivative data representing the dynamics in the derivative space. The LASSO regression then identifies the functions that best describe these dynamics. By limiting the candidate functions to pharmacometrically plausible functions, the proposed structural model is usually reasonable.

The successful identification of functions describing the dynamics learned by the NODE was demonstrated in the weight data example, where the proposed structural model fitted the data comparably well to the NODE. This indicates that the reduction in complexity from a NN to a simpler function did not compromise the model's ability to fit the data. In the two‐compartment PK example, the proposed structural model did not only fit the data well, but its explicit solution is identical to the explicit solution of the central compartment of the conventional two‐compartment PK model. This demonstrates that the proposed structural model describes the same dynamics in the central compartment as the conventional two‐compartment model despite the initial structural difference, i.e., the one‐dimensional character coming from the application of a low‐dimensional NODE. Finally, the warfarin data example illustrates that the approach can derive a structural model that provides a better fit to the data than the traditionally utilized PMX model.

Our approach offers several advantages over other automated modeling approaches for PMX model development.

First, the NNs can freely be placed in the structural model, also in combination with conventional, mechanism‐based model parts. Thus, already known model structures can be defined by the modeler and only unknown dynamics are identified through NNs and LASSO. This was presented in the warfarin example, where the PK part was modeled mechanistically and for the PD part, an indirect response model was defined with only the inhibition of production by warfarin being learned with a NN. Another potential use case for this approach could be to apply NNs only for the absorption if complex absorption processes are observed and the exact mechanistic form is not of primary interest for the modeling question.

Second, the approach is highly flexible and can easily be adjusted by the modeler. On one hand, the candidate functions in the LASSO regression can freely be chosen. In the presented examples, only constant, linear, exponential, and Emax functions were included. However, if the modeler, for example, suspects influence of circadian rhythm on PMX profiles based on pharmacological or physiological knowledge, periodic functions such as sine or cosine can be additionally included as candidate functions. Also, while features are combined additive in the LASSO regression, the modeler can generate features that combine candidate functions multiplicatively if increased flexibility is required to describe the derivative data properly. On the other hand, the identified functions and the resulting proposed structural model can easily be fine‐tuned by the modeler based on pharmacological knowledge in combination with visual inspection of the derivative versus state plots. This means, for example, specific PMX characteristics can be built in, as presented in the warfarin example where the model was rearranged to ensure the steady‐state of PCA if no drug was administered. Further, if the modeler suspects certain parts of the identified set of functions to be artefacts due to outliers or few observations in high or low input‐ranges, such parts can be easily removed or adjusted.

Third, the performance of the presented approach is expected to scale well with model complexity. With the currently applied stepwise modeling approach, more complex dynamics in the PMX data usually require more iterations to identify the structural model that is able to describe these dynamics. However, in the presented approach, the dynamics are automatically learned by the NNs and the LASSO regression identifies the required functions to describe these dynamics without solving any differential equation. Thus, if the NODE fits the data well and the candidate functions for the LASSO‐regression are selected adequately, it is expected that the required resources and time to develop a PMX model with the presented approach are comparable between simple and complex dynamics. However, further analysis is required to quantify the scaling of the presented approach to more complex dynamics.

Fourth, the application of the classical low‐dimensional model structure with state‐ and time‐dependent NNs makes this approach highly suitable for modeling in special populations, such as pediatrics or renally impaired patients. In such special populations, changes in dynamics over time are often observed, for example due to maturation processes in children. Such changes in dynamics are often complex to model. However, they can easily be learned by a time‐dependent NN, and then the LASSO regression can derive the suitable structural model to describe these changes, as presented in the weight model for neonates.

One limitation of the presented approach is the application of low‐dimensional NODEs, i.e., no latent states are modeled with the NNs. This is made obvious in the two‐compartment example, where the model for data generation contained a central and a peripheral compartment but the data was only fitted with a one‐dimensional NODE. However, it has not only been shown that the low‐dimensional NODE approach can fit such data [14], but the explicit solution of the proposed structural model is identical to the two‐compartment model.

A second limitation is that no pharmacological reasoning is directly provided for the proposed structural model. This is evident when looking at the proposed inhibition model for the warfarin PKPD. This differs from the conventionally applied inhibition model for warfarin, which includes only an Emax function that is proposed from ligand‐receptor occupancy dynamics, by an additional exponential function, for which we do not have a pharmacological explanation here. However, the proposed structural model fits the data better in terms of likelihood and BIC. Thus, the presented approach might also be utilized for hypothesis generation. For example, the proposed inhibition model for warfarin may suggest two types of mechanism of action, one described by the Emax function and the other described by the exponential function. In the two‐compartment example, the proposed structural model does not directly provide a model structure with a peripheral compartment for the distribution that would be identified with the standard stepwise model building approach. As mentioned above, this is a consequence of the application of low‐dimensional NODEs without latent states. However, the exponential function can be seen as an increased observed elimination from the central compartment which corresponds to a distribution process until equilibration is reached. The exponential term can even be expressed as a system with a latent compartment, as presented in the Supporting Information. Thus, while no pharmacological, mechanism‐based model is provided directly, it is indicated that two compartments are required to describe the dynamics without explicit time‐dependency.

The presented approach is closely related to the sparse identification of nonlinear dynamics (SINDy) framework [28] where LASSO regression has also been utilized to identify functions in differential equations. One key difference between the presented NODE‐LASSO approach and SINDy is that in the latter approach, derivatives need to be measured or approximated directly from the observed state. This can be challenging for sparse or noisy pharmacometric data, particularly since data is usually measured in multiple subjects with different individual parameters and potentially different dosing regimens. Applying NODEs in the mixed‐effects framework allows approximating derivatives directly from within the differential equation, resulting in clean derivative data. Additionally, the NODE‐LASSO approach allows easily generating derivative data for unknown or complex parts in the model, as has been shown in the warfarin PKPD example.

Overall, the presented automated NODE‐LASSO model development approach based on low‐dimensional NODEs and LASSO regression allows efficient identification of a proposed structural PMX model for PK, PKPD data, and beyond. The proposed structural models may be directly applied to the data as final model or may serve as starting point for further model adjustments and refinements. As such, a combined NODE‐LASSO PMX approach can recover meaningful mechanism‐based structures while reducing the need for extensive iterative model development, highlighting its potential as a resource‐efficient and interpretable modeling strategy in PMX and its applications in model‐informed drug development and clinical research.

## Author Contributions

D.S.B., B. Steiert, B. Steffens, M.P., and G.K. wrote the manuscript, D.S.B. and G.K. designed the research, D.S.B. performed the research, D.S.B., B. Steiert, B. Steffens, M.P. and G.K. analyzed the data.

## Funding

This study was supported by the Swiss National Science Foundation (SNSF) (10003647 and 229140) awarded to G.K.

## Conflicts of Interest

The authors declared no conflicts of interest.

## Supporting information


**Table S1:** Defined range of $u_{E50}$ in the LASSO regression based on approximately observed range of inputs to the NNs in the NODE for the three presented example applications.
**Table S2:** Comparison of estimated and calculated model parameters, respectively, between the proposed model (Equation (20) and (21)) and the explicit two‐compartment model (Equation (22)). Note that parameters for the proposed model were estimated with Equation (20), and parameters AD and BD for Equation (21) were calculated from D, w, k, and p.
**Figure S1:** IWRES vs. Time plots for data from a conventional two‐compartment model with a distribution phase of approximately 6 h with (A) a dosing‐interval of 24 h and (B) a dosing‐interval of 2 h fitted with the proposed structural model. The black line represents a loess‐spline.
**Figure S2:** IWRES vs. Time plots for data from a conventional two‐compartment model with a distribution phase of approximately 6 h with (A) a dosing‐interval of 24 h and (B) a dosing‐interval of 2 h fitted with the adjusted proposed structural model with pseudo‐compartment. The black line represents a loess‐spline.
**Figure S3:** Observation versus prediction plots for the NODE fits of (A) the weight data, (C) the two‐compartment data, and (E) the warfarin PD data, and the corresponding fits with the proposed model in (B) Equation (18), (D) Equation (20), and (F) Equation (24).

**Data S2:** Supporting Information.
