# Supplementary material for: Automated Pharmacometric Model Development by Leveraging Low‐Dimensional Neural ODEs and LASSO Regression
Source: CPT Pharmacometrics Syst Pharmacol. 2026 Jun 24;15(7):e70285. doi: 10.1002/psp4.70285 (PMC13291805; doi:10.1002/psp4.70285)
Supplement: Supplementary file 1 — Table S1: Defined range of uE50 in the LASSO regression based on approximately observed range of inputs to the NNs in the NODE for the three presented example applications. Table S2: Comparison of estimated and calculated model parameters, respectively, between the proposed model (Equation (20) and (21)) and the explicit two‐compartment model (Equation (22)). Note that parameters for the proposed model were estimated with Equation (20), and parameters AD and BD for Equation (21) were calculated from D, w, k, and p. Figure S1: IWRES vs. Time plots for data from a conventional two‐compartment model with a distribution phase of approximately 6 h with (A) a dosing‐interval of 24 h and (B) a dosing‐interval of 2 h fitted with the proposed structural model. The black line represents a loess‐spline. Figure S2: IWRES vs. Time plots for data from a conventional two‐compartment model with a distribution phase of approximately 6 h with (A) a dosing‐interval of 24 h and (B) a dosing‐interval of 2 h fitted with the adjusted proposed structural model with pseudo‐compartment. The black line represents a loess‐spline. Figure S3: Observation versus prediction plots for the NODE fits of (A) the weight data, (C) the two‐compartment data, and (E) the warfarin PD data, and the corresponding fits with the proposed model in (B) Equation (18), (D) Equation (20), and (F) Equation (24). [file PSP4-15-e70285-s001.docx]

**Supplementary material**

**Range of** $\boldsymbol{u}_{\boldsymbol{E}\boldsymbol{50}}$ **for the three presented example applications**

Table S1: Defined range of $u_{E50}$ in the LASSO regression based on approximately observed range of inputs to the NNs in the NODE for the three presented example applications.

| NN | Input range | $\boldsymbol{u}_{\boldsymbol{E}\boldsymbol{50}}$ range |
| --- | --- | --- |
| Characterizing maturation-related weight changes in newborns | | |
| $\boldsymbol{f}_{\boldsymbol{NN}}^{\boldsymbol{W}}$ | $W\in\left[ 3000,4000 \right]_{approx}$ | $3000-4000$ |
| $\boldsymbol{f}_{\boldsymbol{NN}}^{\boldsymbol{t}}$ | $t\in[0,8]$ | $0-8$ |
| Characterizing bi-exponential PK data | | |
| $\boldsymbol{f}_{\boldsymbol{NN}}^{\boldsymbol{C}}$ | $Cc\in\left[ 0,5 \right]_{approx}$ | $0-5$ |
| $\boldsymbol{f}_{\boldsymbol{NN}}^{\boldsymbol{t}}$ | $t\in[0,30]$ | $0-30$ |
| Characterizing PD of Warfarin | | |
| $\boldsymbol{f}_{\boldsymbol{NN}}^{\boldsymbol{C}}$ | $Cc\in\left[ 0,20 \right]_{approx}$ | $0-20$ |

**Comparison of macro parameters derived from estimated micro parameters**

Table S2: Comparison of estimated and calculated model parameters, respectively, between the proposed model (Eq. (20) and (21)) and the explicit two-compartment model (Eq. (22)). Note that parameters for the proposed model were estimated with Eq. (20), and parameters $A_{D}$ and $B_{D}$ for Eq. (21) were calculated from $D$, $w$, $k$, and $p$.

| Model | Parameter | Value |
| --- | --- | --- |
| Proposed model Eq. (20)/(21) | $A'$ | 5.1 |
|  | $p$ | 0.46 |
|  | $B'$ | 5.0 |
|  | $k$ | 0.12 |
| 2-compartment model Eq. (22) | $A$ | 5.1 |
|  | $\alpha$ | 0.46 |
|  | $B$ | 5.0 |
|  | $\beta$ | 0.12 |

**Architecture of NNs utilized in NODEs**

In this manuscript, all NNs consisted of one hidden layer with 5 neurons. The activation function for the hidden neurons was the commonly applied Softplus activation

| $\mathrm{Softplus}\left( x \right)=\frac{1}{\beta}\cdot\log\left( 1+e^{\beta\cdot x} \right)$ | (2) |
| --- | --- |

where the parameter $\beta$ was set to the default in the *pmxNODE* package, i.e., $\beta$ = 20. In NNs depending on time, weights from input- to hidden-layer were restricted to negative values (Bräm, 2023).

**Explicit solution of the proposed structural model for the two-compartment model**

Eq. (20) is a linear non-homogenous differential equation of the form

$$\frac{d}{dt}x\left( t \right)=a\cdot x\left( t \right)+b\left( t \right) , x\left( 0 \right)=x^{0}$$

and has the explicit solution

$$x\left( t \right)=e^{t\cdot a}\cdot x^{0}+\int_{0}^{t} e^{\left( t-\tau\right)\cdot a}\cdot b\left( \tau\right) d\tau.$$

Setting

$a=-k$ , $b\left( t \right)=-D\cdot w\cdot e^{-p\cdot t}$ and $x^{0}=D$

we obtain

$$c\left( t \right)=e^{-k\cdot t}\cdot D-D\cdot w\cdot e^{-k\cdot t}\cdot\int_{0}^{t} e^{k\cdot\tau}\cdot e^{-p\cdot\tau} d\tau$$

$$=e^{-k\cdot t}\cdot D-D\cdot w\cdot e^{-k\cdot t}\cdot\frac{e^{t\cdot(k-p)}-1}{k-p}$$

$$=e^{-k\cdot t}\cdot D-\frac{D\cdot w}{k-p}\cdot\left( e^{-p\cdot t}-e^{-k\cdot t} \right)$$

$$=e^{-k\cdot t}\cdot D+\frac{D\cdot w}{k-p}e^{-k\cdot t}-\frac{D\cdot w}{k-p}e^{-p\cdot t}$$

$$=\frac{D\cdot\left( k-p+w \right)}{k-p}e^{-k\cdot t}-\frac{D\cdot w}{k-p}e^{-p\cdot t} .$$

**Multiple-dose scenario with the proposed two-compartment model**

A previously highlighted limitation of low-dimensional NODEs are multiple-dose scenarios. Since no latent states are included in the low-dimensional NODE, it does not have a “memory” of previous doses. Thus, it was stated that low-dimensional NODEs of form Eq. (1) only are capable of modeling multiple-dose scenarios if the distribution process is finished when the next dose is given, i.e., the follow-up doses are given in the terminal elimination phase, e.g., in a two-compartment model. The same limitation is also applicable for the proposed structural model in Eq. (20). For illustration purpose, data with a two-compartment model was generated where the distribution phase is approximately 6 hours for a multiple-dose scenario with (i) a dosing-interval of 24 hours, i.e., dose was given after distribution phase has finished, and (ii) a dosing-interval of 2 hours, i.e., dose was given within the distribution phase. The proposed model in Eq. (20) was adjusted such that time was time-after-dose according to

|  | $\frac{dC}{dt}=In(D,t)-k\cdot C-D\cdot w\cdot e^{-p\cdot\left( t-t_{D} \right)}, C\left( 0 \right)=0$ |  |
| --- | --- | --- |

where $t_{D}$ is the time of the last dose, $D$ is the amount of the most recent dose, and $In(D,t)$ is the dosing function for a bolus dose. While the proposed model was capable of fitting the data with a dosing-interval of 24 hours, the individual weighted residuals (IWRES) show a structural model misspecification in the scenario with a 2-hours dosing-interval, compare Supplementary Figure S1.

However, the exponential function in the proposed structural model can be described with an additional pseudo-compartment $S$ since


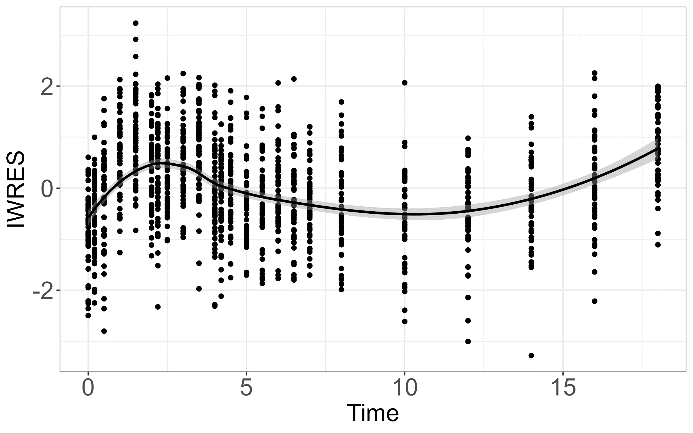

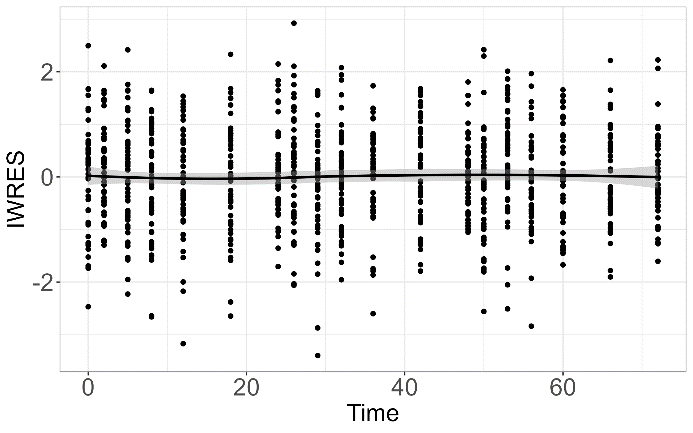


Figure S1: IWRES vs. Time plots for data from a conventional two-compartment model with a distribution phase of approximately 6 hours with A) a dosing-interval of 24 hours and B) a dosing-interval of 2 hours fitted with the proposed structural model. The black line represents a loess-spline.

A)

B)

|  | $D\cdot w\cdot e^{-p\cdot\left( t-t_{D} \right)}=S\left( t \right) with \frac{dS}{dt}=- p\cdot S, P\left( 0 \right)=D\cdot w$ |  |
| --- | --- | --- |

Thus, the proposed structural model can be adjusted according to

|  | $\frac{dC}{dt}=In_{C}\left( D,t \right)-k\cdot C-w\cdot S, C\left( 0 \right)=0$  $\frac{dS}{dt}=In_{P}\left( D,t \right)-p\cdot S, P\left( 0 \right)=0$ |  |
| --- | --- | --- |

where $In_{C}\left( D,t \right)$ is the dosing function for the central compartment, i.e., updating $C$ at a dosing event by adding $D$, and $In_{S}(D,t)$ is the dosing function for the pseudo-compartment, i.e., updating $S$ at a dosing event by adding $D$. With this, the proposed structural model can be adjusted to be also capable of fitting multiple-dose scenarios with shorter dosing-intervals than distribution phases, compare Supplementary Figure S2. However, it is important to note that the pseudo-compartment $S$ does not represent a peripheral compartment with bidirectional distribution dynamics linked to the central compartment. Instead, it reflects the amount of drug removed from the central compartment during the distribution phase, i.e., the apparent increased elimination from the central compartment prior to the onset of the terminal elimination phase.


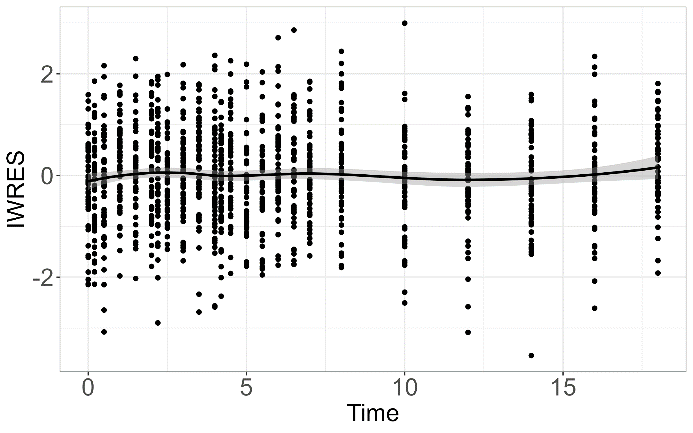

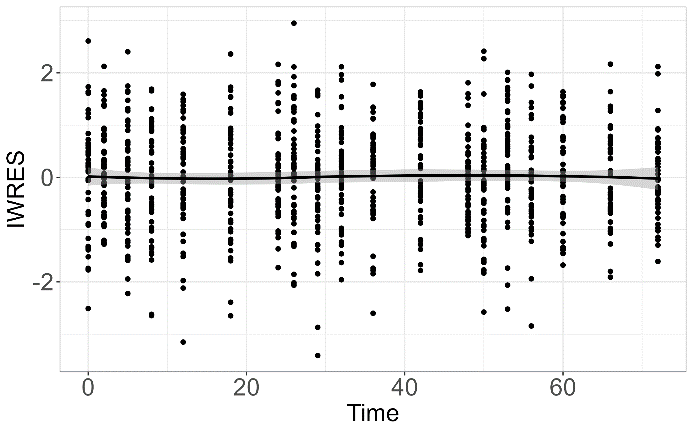


Figure S2: IWRES vs. Time plots for data from a conventional two-compartment model with a distribution phase of approximately 6 hours with A) a dosing-interval of 24 hours and B) a dosing-interval of 2 hours fitted with the adjusted proposed structural model with pseudo-compartment. The black line represents a loess-spline.

A)

B)

**Example model code**

Example model code for $f_{NN}^{t}\left( t \right)$ in the Characterizing maturation-related weight changes in newborns example Eq. (13):

rm(list=ls())

library(tidyverse)

library(glmnet)

#### Derivative data

# Derivatives extracted from the fNN(t) in weight example

derivatives_t <- c(-1,-0.3892,-7e-04,0.1994,0.2877,0.3236,0.3376,0.3429,0.3449,0.3455)

# Times going into the fNN(t) in weight example

states_t <- c(0.1,0.9,1.8,2.7,3.6,4.4,5.3,6.2,7.1,8)

#### Features (only exponential and emax function for simplicity)

# Feature generation for exponential function outputs

exponents <- seq(-2,2,by=0.2)

exp_features <- sapply(exponents,function(x) exp(x * states_t))

# Feature generation for emax function outputs

hills <- seq(0,4,by=0.8)

ec50s <- seq(0,10,by=2.5)

emax_grid <- expand.grid(hills,ec50s)

emax_features <- apply(emax_grid,1,function(x) states_t^x[1]/(x[2]^x[1] + states_t^x[1]))

# Combined features

features <- cbind(exp_features,emax_features)

#### LASSO regression

# LASSO with glmnet; inputs features, target values derivatives

glm_fit <- cv.glmnet(features,derivatives_t)

# Only initercept and 7th feature from exponents selected

# (simplified coefficient selected with "lambda.1se" and not via BIC calculation, due to simplified features)

coef(glm_fit, s="lambda.1se")

# Non-linear fit with derivatives ~ Intercept + exponential function

nls_fit <- nls(derivatives_t ~ Intercept + V * exp(k * states_t),

start = c(Intercept = 0.3, V = -1.4, k = exponents[7]))

# Derivative plot for NN and LASSO

ggplot(data.frame(states = states_t,

derivatives = derivatives_t,

predictions = nls_fit$m$predict())) +

geom_line(aes(x=states,y=derivatives,color="NN"),linewidth=2) +

geom_line(aes(x=states,y=predictions,color="LASSO"), linewidth=2, linetype="dashed") +

scale_color_manual(name = "",

values = c("NN" = "black",

"LASSO" = "red"),

labels = expression("f"[NN]^{phantom(x)*t}*"(t)",

"f"[LASSO]*"(t)"),

breaks = c("NN","LASSO")) +

xlab("Time") +

ylab("Derivatives") +

theme_bw() +

theme(axis.title = element_text(size=20),

axis.text = element_text(size=20),

legend.position = "inside",

legend.position.inside = c(0.8,0.2),

legend.box.background = element_rect(color="black",linewidth = 3),

legend.title = element_blank(),

legend.text = element_text(size=15),

legend.key.height = unit(1,"cm"))

**Observation versus Prediction plots**


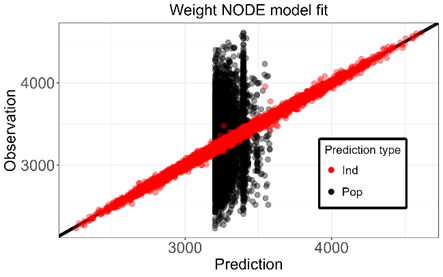

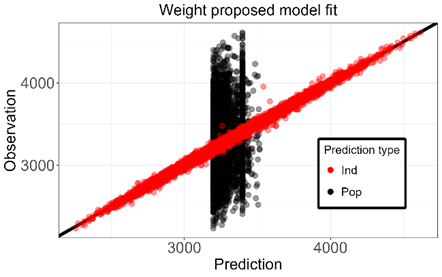


A)

B)


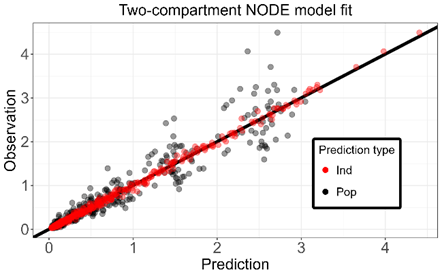

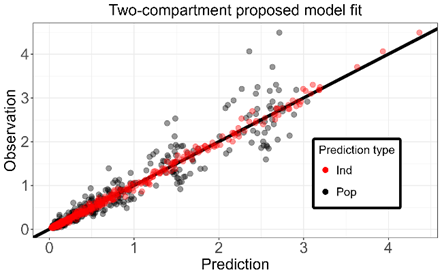


C)

D)


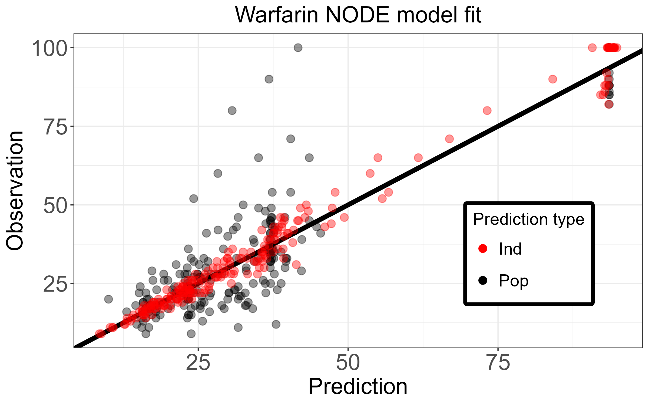


E)


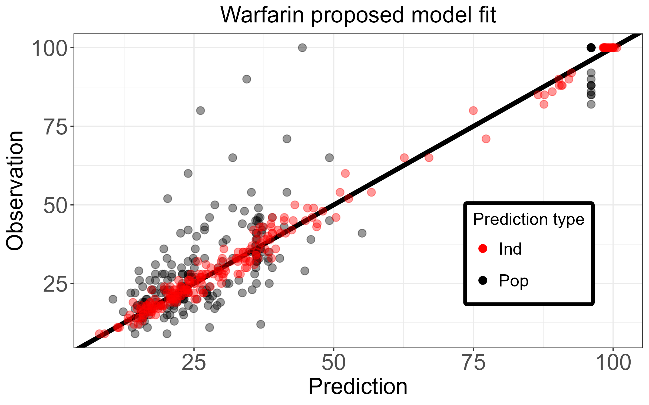


F)

Figure S3: Observation versus prediction plots for the NODE fits of A) the weight data, C) the two-compartment data, and E) the warfarin PD data, and the corresponding fits with the proposed model in B) Eq. (18), D) Eq. (20), and F) Eq. (24).
